# Effective Cycle Slip Detection and Repair for PPP/INS Integrated Systems

**DOI:** 10.3390/s19030502

**Published:** 2019-01-25

**Authors:** Sheng Yang, Leilei Li, Jingbin Liu, Qusen Chen, Xuewen Ding, Hongxing Sun, Yu Wu, Chunhua Ren, Ning Hu

**Affiliations:** 1 State Key Laboratory of Information Engineering in Surveying, Mapping and Remote Sensing, Wuhan University, Wuhan 430079, China; shengy@whu.edu.cn (S.Y.); jingbin.liu@whu.edu.cn (J.L.); sunhx@whu.edu.cn (H.S.); 2 Key Laboratory of Optoelectronic Technology and Systems of the Ministry of Education of China, Chongqing University, Chongqing 400044, China; rchht@163.com; 3 College of Aerospace Engineering, Chongqing University, Chongqing 400044, China; cquwuyu@cqu.edu.cn (Y.W.); ninghu@cqu.edu.cn (N.H.); 4 Collaborative Innovation Center of Geospatial Technology, Wuhan University, Wuhan 430079, China; 5 Department of Remote Sensing and Photogrammetry and the Center of Excellence in Laser Scanning Research, Finnish Geospatial Research Institute, Masala 02430, Finland; 6 School of Geodesy and Geomatics, Wuhan University, Wuhan 430079, China; chenqs@whu.edu.cn; 7 Qianxun Spatial Intelligence Inc, Shanghai 200438, China; xwding9@163.com

**Keywords:** PPP, INS, cycle slip, detection and repair

## Abstract

Cycle slip (CS) is a primary error source in Precise Point Positioning/Inertial Navigation System (PPP/INS) integrated systems. In this study, an INS-aided CS detection and repair method is presented. It utilizes high-precision INS information instead of a pseudorange to remove the satellite–receiver geometric range in the wide-lane (WL) and ionospheric-free (IF) phase combinations and creates an INS-aided WL (WL-INS) model and an INS-aided IF (IF-INS) model. Since INS information is superior to pseudorange, the INS-aided models have high detection accuracy. However, the effectiveness of INS-aided models cannot persist for a long time because of INS accumulation error. To overcome the disturbance of INS error, improved INS-aided models are proposed. This idea takes advantage of the long wavelength of WL combination and tries to fix WL CS. Once it succeeds, the INS error can be evaluated and removed. The proposed method was tested using land vehicle data, in which simulated cycle slips and signal interruption were introduced. The results show that this method can accurately detect and repair different cycle slips between the continuous Global Positioning System (GPS) epoch. When it comes to the cycle slip after a GPS interruption, the method can also accelerate PPP re-convergence, as it is not affected by the inertial accumulation error.

## 1. Introduction

Precise Point Positioning (PPP) has received extensive attention over the last decade because it provides high-precision positioning with only a single receiver. Combined with an Inertial Navigation System (INS), the PPP/INS can provide both positioning and orientation information with a high sampling rate. PPP and its integration system are suitable for applications where differential positioning is not applicable, such as disaster assessment and management, marine surveying and environmental monitoring in isolated areas [1,2,3]. However, PPP can be easily deteriorated by a cycle slip (CS) or signal loss of lock due to high dynamics, low satellite elevation, or obstruction along the signal path. It needs at least 10 min for re-convergence, which is disastrous and unacceptable to the task.

Extensive studies have been conducted on cycle slip detection and repair in recent years. These methods can generally be classified into the following four models [4]: undifferenced, double-differenced, triple frequency, and inertial-aided. Bisnath and Langley proposed an automated cycle slip detection method with double-differenced geometry-free linear combinations [5]. Chen et al. utilised double-differenced geomerty-free and IF observation to estimate the cycle slips in dual-frequency observations [4]. A real-time cycle slip detection using triple-frequency observations was also developed by Lacy et al. and Zhao et al. [6,7]. However, the double-differenced model requires carrier phase observation on the base station and is unsuitable for PPP. Moreover, the triple frequency model is not popular in current practical applications as most receivers cannot support the third frequency signal. Therefore, undifferenced and inertial-aided models are two applicable solutions to the cycle slip problem in PPP.

Blewitt made the first effort in the undifferenced model using the TurboEdit method, which was defined under the condition of the P-code pseudorange and smoothly varying ionospheric electron content [8]. This algorithm has been widely applied in high precision Global Navigation Satellite System (GNSS) software, such as GIPSY [9], BERNESE [10], and PANDA [11]. Liu and Cai et al. formulated an improved TurboEdit algorithm that worked well during periods of strong ionospheric scintillation [12,13]. Furthermore, De Lacy et al. introduced Bayesian theory to deal with the problem of detecting and correcting cycle slips [14]. Banville and Langley proposed a cycle slip instantaneous correction method, where the cycle slip parameters were introduced to least-square adjustment [15]. Geng et al. precisely predicted ionospheric delays to accelerate ambiguity resolution [16]. Some of the above methods employed the Melbourne–Wübbena (MW) combination, which is effective to detect large cycle slips. However, the MW combination is vulnerable to code observation noise and unsuitable for small cycle slip detection.

In recent years, INS-aided methods have emerged to solve the cycle slip problem for PPP. Du and Gao used INS-aided wide-lane (WL) and extra-WL phase combination to determine the cycle slips in the L1 and L2 frequencies [3]. Kim et al. proposed an inertial-aided cycle slip detection method that took the satellite geometry into account [17]. Their methods are similar to the idea of cycle slip detection in differential Global Positioning System (GPS)/INS integration proposed by Colombo et al. [18], Altmayer [19] and Lee et al. [20], which uses the INS position to check the cycle slips. As long as the error model of the inertial measurement unit (IMU) is calibrated by PPP/INS integrated systems, the position of INS will have very good short-term accuracy. Nevertheless, these methods are subjected to the influence of inertial accumulation error. They can only maintain good accuracy for continuous observations or GPS signal short-term loss. If the signal interruption continues to extend, the influence of the inertial accumulation error cannot be ignored and those methods will fail to work. An alternative approach to defuse the inertial accumulation error is using high-level IMU sensors, but the accuracy of an inertial sensor is directly related to its cost.

Therefore, we present an effective INS-aided cycle slip detection and repair method for PPP/INS integration, which is immune to the INS accumulation error. The basic idea is that once the cycle slip on WL is fixed, the inertial accumulation error can be evaluated and removed from the INS-aided models. This approach utilizes high-precision INS information to remove the satellite–receiver geometric range in WL and ionospheric-free (IF) phase combinations. The satellite-difference and epoch-difference processing are implemented to eliminate the receiver clock bias, satellite clock bias, and atmospheric delays. When WL CS is fixed, two INS error-free CS detection models are consequently created with relatively long-term high precision. And the cycle slips on L1 and L2 can be determined by joint solution of the two INS error-free models. Experimental results indicate that the proposed INS error-free models expand the effectiveness duration of INS-aided methods. This can detect and repair cycle slips, and accelerate the re-convergence of a PPP solution even after a long interruption of GPS signal. This method is also very suitable for real-time cycle slip detection and repair because it only uses previous and current epoch observations and does not require time smoothing, which is required by the TurboEdit method.

In the following section, PPP/INS integration is introduced, followed by the methodology of detecting and repairing cycle slip with combined observations from WL-INS and IF-INS model. The INS error-free models are then presented to remove INS error and raise the precision of cycle slip determination. Numerical experiments are presented and discussed to evaluate the performance of the proposed method. The conclusions are provided in the final section.

## 2. Precise Point Positioning/Inertial Navigation System (PPP/INS) Integration

Assuming that the satellite orbit and clock errors are removed by applying the precise satellite orbit and clock products, and other system errors including the antenna phase center offset, phase wind-up effect, relativistic effect, earth tides, and ocean loading are corrected, the carrier phase measurements can be expressed as follows:

(1) $$L_{i}=\rho +cdt_{r}+T-I_{i}+\lambda_{i}N_{i}+\varepsilon \left(L_{i}\right)$$

where L is the phase observation, ρ is the geometric distance between the satellite and receiver, c is the speed of light, $dt_{r}$ is the receiver clock bias, T is a slant tropospheric delay, I is a slant ionospheric delay, N and λ are float ambiguity and wavelength, respectively, ε is the unmodeled error, including observation noise and multipath effect, and *i* denotes the frequency of the satellite signal.

To eliminate the ionospheric effects in the signal measurements, an IF linear combination is often utilized in PPP data processing, which can be expressed as:

(2) $$L_{IF}=\rho +cdt_{r}+T+\lambda_{IF}N_{IF}+\varepsilon \left(L_{IF}\right)$$

where $L_{IF}$ is the ionospheric-free phase observation, $N_{IF}$ and $\lambda_{IF}$ are the ionospheric-free ambiguity and wavelength, respectively.

**Figure 1:**
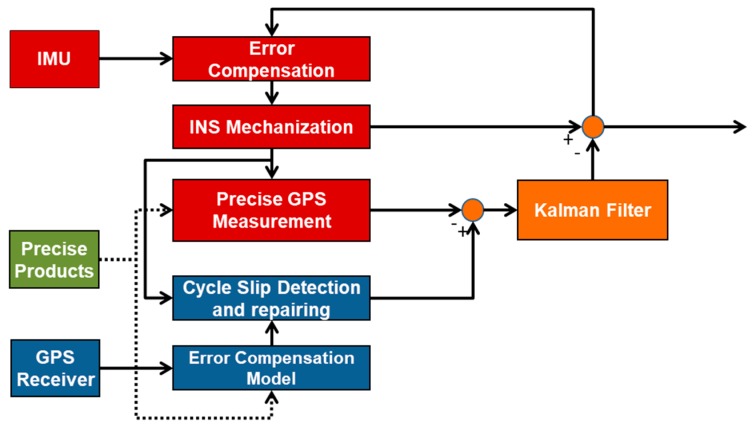
Architecture of Precise Point Positioning/Inertial Navigation System (PPP/INS) tightly integrated system.

Compared with PPP used alone, PPP/INS integration provides higher data rates to find position. It also helps fill in gaps in the case of PPP signal loss of lock. The integration can be divided into two basic modes, i.e., loosely and tightly coupling modes. Although the cycle slip detection and repair method presented in this research can be implemented in both modes, only the second model will be briefly discussed. The tightly coupling mode uses a Kalman filter to fuse the original observation of PPP and resolution via INS mechanization, as illustrated in Figure 1. The resulting error estimates are sent to the INS to find the filtered positioning and orientation output. Meanwhile, the INS outputs are also used to assist PPP in cycle slip detection and repair to enhance the quality control of the integrated system. Considering the system performance and observability factors, a Kalman filter with 15 INS error states plus GPS states was developed in our processing software. The filter state vector is given by:

(3) $$x={\left(R_{INS},V_{INS},\Phi_{INS},b_{acc},b_{gyro},dt,d\dot{t},T,N_{IF,1},N_{IF,2}\ldots ,N_{IF,n}\right)}^{T}$$

where the states are INS position error, INS velocity error, INS attitude error, accelerometer bias, gyro bias, receiver clock bias, receiver clock drift, zenith tropospheric wet delay, and IF ambiguities in sequence. The Kalman filter measurements are the differences among the INS estimated phase, pseudorange, Doppler, and the corresponding GPS observations.

## 3. INS-Aided Cycle Slip (CS) Detection and Repair

The main idea of INS-aided CS detection and repair in PPP/INS is to create a CS-sensitive detection and repair model. This goal is achieved by removing satellite–receiver geometric range in phase observation with high-precision INS information instead of pseudorange observation. It is also conducted in cooperation with other error elimination methods, i.e., satellite differencing, epoch differencing, ionospheric delay model, and linear combination of observations. The ultimate aim is to speed up the re-convergence of PPP when signal discontinuity occurs. The details of the INS-aided method will be discussed in the following three parts: WL-INS CS detection model, IF-INS CS detection model, INS-aided CS repair and ambiguity recovery method.

### 3.1. Wide-Lane (WL)-INS Model

From Equation (1), a WL measurement equation can be formed as follows:

(4) $$L_{WL}=\rho +cdt_{r}+T-I_{WL}+\lambda_{WL}N_{WL}+\varepsilon \left(L_{WL}\right)$$

where the subscript WL denotes the wide-lane combination with a long wavelength and can be detected easily. A detailed procedure for INS-aided WL detection is as follows:

Firstly, the satellite–receiver geometric range ρ can be predicted by the INS position and precise satellite orbit product.

(5) $$\rho =\rho_{INS}+\varepsilon_{INS}=\sqrt{{\left(X^{s}-X_{INS}\right)}^{2}+{\left(Y^{s}-Y_{INS}\right)}^{2}+{\left(Z^{s}-Z_{INS}\right)}^{2}}+\varepsilon_{INS}$$

where $\rho_{INS}$ is the predicted satellite–receiver geometric range and is based on the INS position and the satellite position, $\left(X_{INS},Y_{INS},Z_{INS}\right)$ is the INS position, $\left(X^{s},Y^{s},Z^{s}\right)$ is the satellite position, and $\varepsilon_{INS}$ represents the error introduced by the INS, i.e., the INS-predicted geometric range error (abbreviated as INS error in the following section). Because the INS has excellent short time performance, the predicted satellite–receiver geometric range is very accurate.

By substituting Equation (5) into Equation (4), the INS-aided WL combination $L_{WL-INS}$ can be created as follows:

(6) $$L_{WL-INS}=L_{WL}-\rho_{INS}=cdt_{r}+T-I_{WL}+\lambda_{WL}N_{WL}+\varepsilon \left(L_{WL}\right)+\varepsilon_{INS}$$

Subsequently, satellite differential processing is implemented by selecting an appropriate reference satellite to eliminate the receiver clock error. The satellite differential processing also removes other receiver errors, such as the uncalibrated phase delay.

(7) $$\Delta L_{WL-INS}=\Delta T-\Delta I_{WL}+\lambda_{WL}\Delta N_{WL}+\Delta \varepsilon \left(L_{WL}\right)+\Delta \varepsilon_{INS}$$

where Δ indicates the differencing operation between satellites.

Finally, epoch differential processing is implemented to the observations. For a low dynamic receiver, the tropospheric delay varies slightly in a few minutes under stable meteorology [21,22,23]. Therefore, the remaining tropospheric delay can be ignored after applying parameter estimation and epoch difference. Ionospheric delay usually has a strong temporal correlation over a few minutes [24,25,26,27]. In the case of an outage of several minutes, centimeter-level prediction accuracy can be obtained by applying an ionospheric fitting model [16,28]. Thus, the effect of ionospheric delay can also be eliminated by the ionospheric fitting model and epoch differential processing. The double differenced WL-INS model between two satellites and two consecutive epochs, i.e., satellite-epoch differenced WL-INS model, can be represented as follows:

(8) $$\delta \Delta L_{WL-INS}=\lambda_{WL}\delta \Delta N_{WL}+\delta \Delta \varepsilon \left(L_{WL}\right)+\delta \Delta \varepsilon_{INS}$$

(9) $$\delta \Delta N_{WL}=\delta \Delta N_{1}-\delta \Delta N_{2}$$

where δ is the epoch differencing operator, $\lambda_{WL}\delta \Delta N_{WL}$ is the satellite-epoch differenced WL CS in meter units, and $\delta \Delta \varepsilon \left(L_{WL}\right)$ and $\delta \Delta \varepsilon_{INS}$ are satellite-epoch differenced WL phase noise and INS error, respectively.

In general, the carrier phase measurement error is about 3 mm. As a consequence, the satellite-epoch differenced WL error $\delta \Delta \varepsilon \left(L_{WL}\right)$ is much smaller than the WL wavelength. Epoch differencing eliminates the common positioning error of INS between two adjacent epochs. Only the accumulated INS error in the time interval remains, which depends on the IMU accuracy level and the sampling rate. For a short time interval, the INS accumulation error is limited, and $\delta \Delta L_{WL-INS}$ has a short-term high accuracy for cycle slip detection. Therefore, the WL-INS model is sensitive to small cycle slip and unaffected by positioning bias when PPP does not reach the convergence. However, it cannot detect equal cycle slips on dual frequencies. To avoid missing reports of cycle slip, it must be combined with other cycle slip methods.

### 3.2. Ionospheric-Free (IF)-INS Model

Following the same procedure described in the section of WL-INS model, the IF-INS CS detection model can be established as Equation (10). When the INS and a precise ephemeris are used to provide high-precision position information for receivers and satellites, the range information in IF combination will be accurately estimated. Subsequently, the receiver clock error will be removed by satellite differential processing, and the tropospheric delay and ambiguity parameters will be eliminated by epoch differential processing.

(10) $$\delta \Delta L_{IF-INS}=\lambda_{IF}\delta \Delta N_{IF}+\delta \Delta \varepsilon \left(L_{IF}\right)+\delta \Delta \varepsilon_{INS}$$

(11) $$\lambda_{IF}\delta \Delta N_{IF}=\alpha \lambda_{1}\delta \Delta N_{1}-\beta \lambda_{2}\delta \Delta N_{2},\alpha =\frac{f_{1}^{2}}{f_{1}^{2}-f_{2}^{2}}，\beta =\frac{f_{2}^{2}}{f_{1}^{2}-f_{2}^{2}}$$

where $\lambda_{IF}\delta \Delta N_{IF}$ is the satellite-epoch differenced IF CS in meter units, $\delta \Delta \varepsilon \left(L_{IF}\right)$ is the satellite-epoch differenced IF phase noise, and $\delta \Delta \varepsilon_{INS}$ is the satellite-epoch differenced INS error. Similarly, both satellite-epoch differenced IF error $\delta \Delta \varepsilon \left(L_{IF}\right)$ and INS error $\delta \Delta \varepsilon_{INS}$ are small enough to ensure good detection accuracy in a short period. The IF-INS model is unaffected by the dramatic changes in the ionosphere. However, it cannot detect the special pairs of cycle slips, such as 60/77. In this research, we propose the INS-aided method that combines the WL-INS model and IF-INS model to detect and repair cycle slips in dual-frequency observations.

### 3.3. INS-Aided CS Repair and Ambiguity Recovery

On the basis of the WL-INS CS detection model $\delta \Delta L_{WL-INS}$ and IF-INS CS detection model $\delta \Delta L_{IF-INS}$, the cycle slips in the L1 and L2 frequencies can be easily determined by the following equation:

(12) $$\{\begin{array}{c} \delta \Delta {\tilde{N}}_{1}=\frac{\beta \lambda_{2}*\delta \Delta L_{WL-INS}/\lambda_{WL}-\delta \Delta L_{IF-INS}}{\beta \lambda_{2}-\alpha \lambda_{1}}\\ \delta \Delta {\tilde{N}}_{2}=\frac{\alpha \lambda_{1}*\delta \Delta L_{WL-INS}/\lambda_{WL}-\delta \Delta L_{IF-INS}}{\beta \lambda_{2}-\alpha \lambda_{1}} \end{array}$$

where $\delta \Delta {\tilde{N}}_{1}$ and $\delta \Delta {\tilde{N}}_{2}$ are the real cycle slips on L1 and L2 frequencies, respectively.

The strategy of rounding recovery is applied to the INS-aided CS repair and ambiguity recovery. It rounds the combined real solution of the WL-INS model and IF-INS model. If the rounding error is small enough and the rounded cycle slips pass the validation checks, the ambiguities are successfully recovered. Otherwise, the real cycle slips are added to the ambiguities at a previous epoch, which are treated as the initial values of PPP convergence. Compared with the code-assisted ambiguity recovery method, the INS-aided solution has a better short-term accuracy and makes it easier to repair cycle slips or shorten the time of ambiguity re-convergence.

However, INS drift will be unneglectable when the GPS signal is interrupted for a long time, and the WL-INS and IF-INS model will become invalid. Therefore, reducing the influence of INS accumulation error becomes the critical problem to apply the INS-aided method in relatively long GPS interruption. An alternative approach is the use of high-level IMU sensors, but it is costly. Here, we propose the INS error-free models by taking advantage of the easily fixing characteristic of WL CS. The improved cycle slip detection models are still INS-aided but enhanced with the feature of INS error-free, thereby resulting in improved performance when long GPS interruption occurs.

## 4. INS Error-Free WL-INS and IF-INS Model

As the INS-aided models depend significantly on the accuracy of INS, the cycle slip will fail to be detected if INS error goes too large. This section mainly describes the method of estimating and removing the INS error of the WL-INS and IF-INS models. It takes advantage of the long wavelength of WL combination and tries to fix the cycle slip on WL. Once it succeeds, the INS error in the wide-lane and ionosphere-free detection models are evaluated, and the INS error-free models are consequently produced with an improved performance.

### 4.1. Error Evaluation

The WL satellite-epoch differenced CS is easy to fix when using the Least-square Ambiguity Decorrelation Adjustment (LAMBDA) method or other methods because WL phase combination has a wavelength of 86 cm. Once the WL CS is fixed, the error term, including satellite-epoch differenced WL phase observation error and INS error, can be determined. For simplicity, we use the rounding method to obtain the WL CS. From Equation (8), the fixed WL satellite-epoch differenced CS can be expressed as:

(13) $$\delta \Delta {{\tilde{N}}^{\prime }}_{WL}=\left[\frac{\delta \Delta L_{WL-INS}}{\lambda_{WL}}\right]$$

where [ ] denotes the rounding operator. By substituting Equation (13) into Equation (8), the residual error of WL-INS model, i.e., the sum of the satellite-epoch differenced phase observation error and INS error, can be obtained as follows:

(14) $$\delta \Delta \varepsilon_{INS}+\delta \Delta \varepsilon \left(L_{WL}\right)=\delta \Delta L_{WL-INS}-\delta \Delta {{\tilde{N}}^{\prime }}_{WL}*\lambda_{WL}$$

where the satellite-epoch differenced INS error and phase error cannot be separated. For long-term interruption, the INS error is much larger than the WL phase error and dominates the residual error stated in the left of Equation (14). 

### 4.2. INS Error-Free Models

When the cycle slip on WL is fixed, the residual error can be removed from the WL-INS model, and the INS error is also excluded from the model as a consequence. The INS error-free WL-INS model can be expressed as follows:

(15) $$\delta \Delta {L^{\prime }}_{WL-INS}=\lambda_{WL}\delta \Delta {{\tilde{N}}^{\prime }}_{WL}$$

By substituting the residual error of Equation (14) into Equation (10) and shifting the IF and WL phase errors to the right side of the equation yields, the INS error-free IF-INS model can be obtained as follows:

(16) $$\begin{array}{l} \delta \Delta {L^{\prime }}_{IF-INS}=\delta \Delta L_{IF-INS}-\delta \Delta L_{WL-INS}+\delta \Delta {{\tilde{N}}^{\prime }}_{WL}*\lambda_{WL}\\ =\lambda_{IF}\delta \Delta N_{IF}+\delta \Delta \varepsilon \left(L_{IF}\right)-\delta \Delta \varepsilon \left(L_{WL}\right) \end{array}$$

Compared to the IF-INS model in Equation (10), Equation (16) introduces the WL phase error instead of the INS error. Strictly, what we can determine from Equation (14) is the sum of phase observation error and INS error. Nevertheless, the phase observation error can always be ignored when compared to the INS error. When WL CS is fixed, two INS error-free CS detection models are consequently created without the $\delta \Delta {L^{\prime }}_{WL-INS}$ influence of inertial accumulation error. Based on the INS error-free WL-INS model $\delta \Delta {L^{\prime }}_{WL-INS}$ and INS error-free IF-INS model $\delta \Delta {L^{\prime }}_{IF-INS}$, the cycle slips $\delta \Delta {{\tilde{N}}^{\prime }}_{1}$ and $\delta \Delta {{\tilde{N}}^{\prime }}_{2}$ in the L1 and L2 frequencies can also be determined similar to Equation (12).

Cycle slip repair is highly dependent upon the accuracy of calculated WL CS. In general, the WL CS $\delta \Delta {{\tilde{N}}^{\prime }}_{WL}$ can be determined correctly. Once $\delta \Delta {{\tilde{N}}^{\prime }}_{WL}$ is fixed, the INS error can be evaluated and removed from both the WL-INS and IF-INS model. Although the satellite-epoch differenced WL and IF phase error remain in the real cycle slips, they will be equally distributed to cycle slips on L1 and L2, i.e., $\delta \Delta \varepsilon \left(L_{1}\right)=\delta \Delta \varepsilon \left(L_{2}\right)$, with the magnitude not exceeding one or two cycles. These errors will cause a bias of 0.1–0.2 m on IF ambiguities, which are much lower than the code-aided method. INS error-free strategy extends the INS-aided method to situations where long-term interruption occurs or low-cost Micro-Electromechanical Systems (MEMS) IMU is applied.

## 5. Numerical Experiments and Analyses

**Figure 2:**
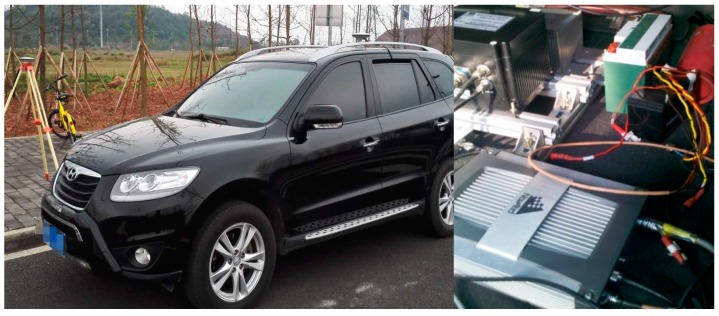
The research set and the area of the field test.

Field test data were collected by a land vehicle on an open area in the suburbs of Beijing, China. A NovAtel SPAN-SE dual-frequency GPS receiver and a tactical-grade IMU were mounted in the trunk, while the GPS antenna was mounted on the roof of the vehicle. The IMU consists of three closed-loop fiber optic gyroscopes and three quartz accelerometers with bias stability of 0.3°/h and 0.1 mg, respectively. The sampling rate of GPS data was 1 Hz, whereas the IMU measurements were recorded at a rate of 200 Hz. Figure 2 shows the research set and the area of the field test. Waypoint Inertial Explorer post-processing software was used to calculate the reference trajectory of the test.

**Figure 3:**
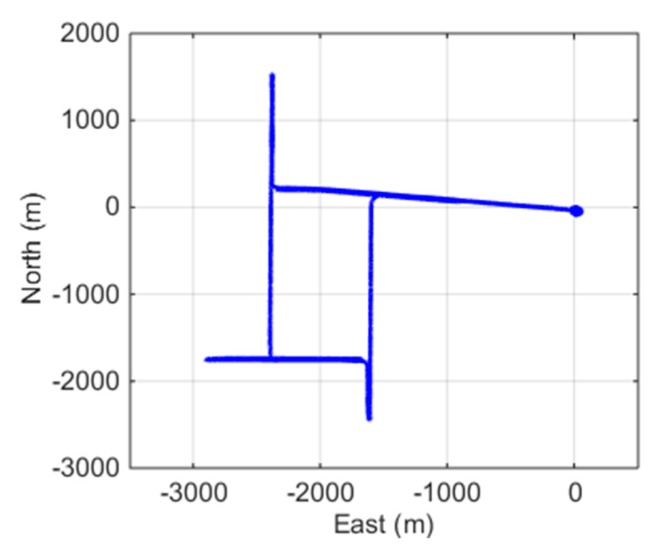
Trajectory of the land vehicle.

The whole duration of the dataset was 1.5 h, and the vehicle ran for about 15 km at a speed of up to 35 km/h. To initialize the integration system, the vehicle stopped for about 15 min before driving away. It also came across a few traffic red lights during driving. The trajectory of the test is shown in Figure 3. The PRN 03 was chosen as the reference satellite because it had a high elevation angle above 70° throughout the test, and no cycle slips occurred.

### 5.1. INS-Aided CS Detection and Repair

**Figure 4:**
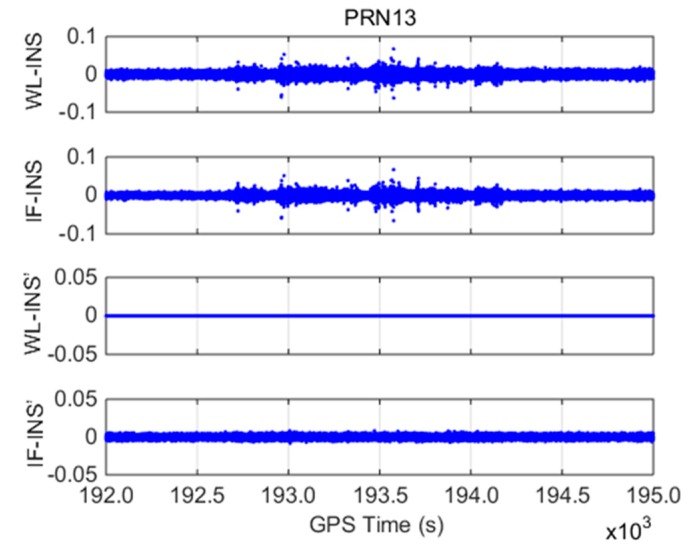
Cycle slip (CS) detection models for PRN13 (unit: meter).

With PRN 13 as an example, INS-aided models, including WL-INS model $\delta \Delta L_{WL-INS}$, IF-INS model $\delta \Delta L_{IF-INS}$, INS error-free WL-INS model $\delta \Delta {L^{\prime }}_{WL-INS}$, and INS error-free IF-INS model $\delta \Delta {L^{\prime }}_{IF-INS}$, are shown in Figure 4.

(1) The amplitudes of $\delta \Delta L_{WL-INS}$ and $\delta \Delta L_{IF-INS}$ are within ±0.1 m, and the standard deviations are 0.0058 and 0.0049 m, respectively. The two models show similar processes over time because they both contain the INS error, which is the main error in the detection models. 

(2) For the INS error-free models, $\delta \Delta {L^{\prime }}_{WL-INS}$ does not contain the error term once WL CSs are correctly fixed. Therefore, its value is zero throughout the test. The value of $\delta \Delta {L^{\prime }}_{IF-INS}$ is within ±0.01 m, and the standard deviation is 0.002 m because $\delta \Delta {L^{\prime }}_{IF-INS}$ contains WL and IF phase observation error. The detection accuracy of the INS error-free models is greatly improved compared with that of the WL-INS and IF-INS models. During the whole test period, the detection models of PRN 13 do not change significantly, which indicates that cycle slips not occur.

**Figure 5:**
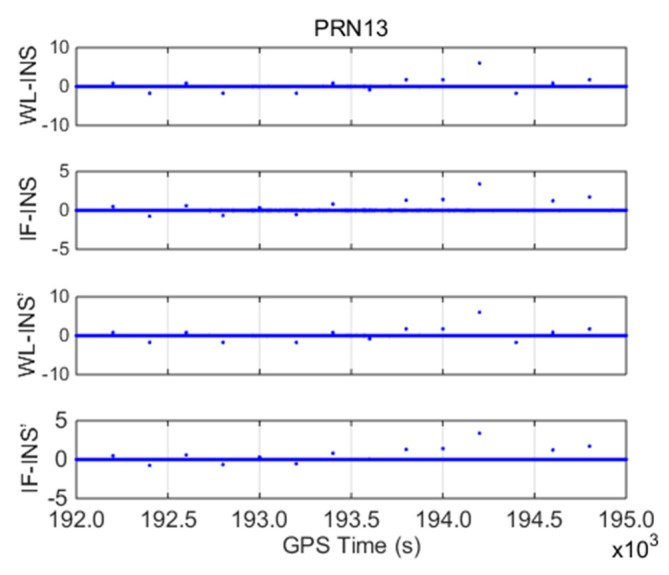
Cycle slip detection models for PRN13 with simulated cycle slips (unit: meter).

To evaluate the performance of the INS-aided method, simulated cycle slips were manually introduced to the carrier phase observations of PRN 13. They had an equal time interval of 200 s with a total number of 14 pairs, including small cycle slips, special pairs cycle slips, and equal cycle slips on L1 and L2 frequencies. Table 1 summarizes the simulated cycle slip values, corresponding detection models, and real cycle slip solutions using the proposed method. Figure 5 shows the four cycle slip detection models for the whole duration of the test.

Considering the impact of various errors, the thresholds of $\delta \Delta L_{WL-INS}$, $\delta \Delta L_{IF-INS}$, and $\delta \Delta {L^{\prime }}_{IF-INS}$ were set to 0.3, 0.3, and 0.05 m, respectively. The following conclusions can be drawn from this study.

(1) The WL-INS model is highly sensitive to small WL CSs, but it cannot detect the equal cycle slips on L1 and L2 frequencies (such as 3/3). The IF-INS model can detect small cycle slips and equal cycle slips on dual frequencies, but it is insensitive to the special pairs cycle slips (such as 4/5 and 7/9). Therefore, through the joint use of the WL-INS and IF-INS models, all 14 pairs of cycle slips can be successfully detected. The combination of the INS error-free WL-INS and IF-INS model also achieves similar detection accuracy.

(2) Given that the PPP measurements are collected at 1 Hz, the inertial accumulation error is minimal during this period. Hence, both the INS-aided models and the INS error-free models can successfully solve the integer cycle slips with the rounding precision within ±0.1 cycles.

### 5.2. INS-Aided Ambiguity Recovery

When GPS signal is interrupted, the ambiguities usually need to be reinitialized. In this study, we use the INS-aided method to determine cycle slips after the interruption. If the obtained cycle slips are correct, the ambiguities can be successfully recovered within a single epoch. Otherwise, cycle slips are added to the ambiguities before the interruption, which are treated as the initial value of PPP convergence.

To evaluate the long-term performance of the proposed INS-aided method, simulated signal interruptions with different durations (10–60 s with 10 s as an increment step) were introduced to the data, as well as simulated cycle slips on all satellites (except the reference satellite). Cycle slips differ with satellites but cover large, small, special pairs, and equal cycle slips on dual frequencies (as shown in Table 2). By applying the INS-aided CS detection models and INS error-free models to the simulated data, we have the resolution of cycle slips (Table 2).

(1) The INS-aided CS detection models can successfully detect and repair cycle slips when GPS outage is within 20 s. However, if the outage is continuous, the INS-aided method decreases in accuracy significantly and will finally lose its effectiveness because of inertial accumulation error.

(2) For the INS error-free models, the INS accumulation error is eliminated by fixing WL CSs. When PPP data are interrupted for 50 s or less, the cycle slips on all satellites can still be successfully detected and repaired even though the rounding errors are apparently increasing as the outage increasing. When the outage reaches 60 s, one satellite cycle slip is resolved with an incorrect value, and others are also with excessive residual error. However, as the WL CSs are successfully fixed, the remaining errors are equally distributed to cycle slips on L1 and L2 with one cycle magnitude. 

**Figure 6:**
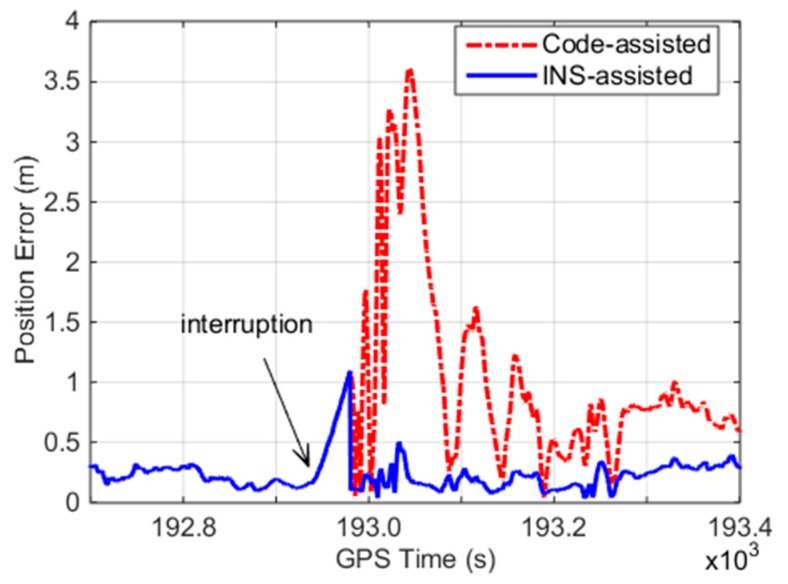
Position error for the INS-assisted ambiguity recovery method and the code-assisted method.

For the outage of 60 s, we obtain the real cycle slips from the float solution of the INS error-free method. These cycle slips are then combined with the ambiguities before the interruption as the initial ambiguities for PPP re-convergence. The positioning error of the INS error-free models is calculated with the reference of differential GPS solution. In addition, code-assisted ambiguity recovery is applied to the data for comparison, as depicted in Figure 6. The INS error-free models decrease the positioning error and accelerate the re-convergence time of PPP compared with the code-assisted method. Although a pair of incorrect cycle slips by the INS error-free method exists, it only causes 0.1–0.2 m ambiguity range errors in the corresponding satellite, which are much smaller than those caused by the code-assisted method.

## 6. Conclusions

In this work, an effective INS-aided CS detection and repair method is proposed for PPP/INS integration to speed up the convergence of PPP solution. It utilizes high-precision INS and a precise ephemeris instead of pseudorange to obtain the satellite–receiver geometric distance. Therefore, it has an excellent performance for cycle slips between continuous GPS epochs. Unlike other existing INS-aided cycle slip detection methods, the presented method evaluates and removes the inertial accumulation error from the cycle slip detection models by fixing WL CSs. It can recover the ambiguity within one epoch after a long-term GPS interruption. The presented method was tested using the land vehicle data, where different kinds of simulated cycle slips and signal interruptions were introduced. It shows adaptability when encountering large, small, special pairs, or equal cycle slips on L1 and L2 frequencies, and can speed up the convergence of PPP solutions even encountering 1 min GPS signal interruption when a tactical grade IMU is used. 

In addition, the INS-aided method has some attendant merits. It does not require time smoothing, which is required by the TurboEdit method. Only previous and current epoch observations are needed for processing. Therefore, this method is suitable for real-time applications. It also makes it possible to use low-cost MEMS IMU in PPP/INS integration since the regulation of the INS error is loosened. Epoch differencing eliminates the common positioning error between neighbor epochs, so this method is effective even when PPP does not reach the convergence.

## Figures and Tables

**Table 1:** Simulated cycle slip values, corresponding detection models, and real cycle slip solutions for PRN13.

Global Positioning System (GPS) Time (s)	Simulated L1 CS (cycle)	Simulated L2 CS (cycle)	$\delta \Delta L_{WL-INS}(m)$	$\delta \Delta L_{IF-INS}(m)$	$\delta \Delta {\tilde{N}}_{1}(\mathrm{cycle})$	$\delta \Delta {\tilde{N}}_{2}(\mathrm{cycle})$	$\delta \Delta {L^{\prime }}_{WL-INS}(m)$	$\delta \Delta {L^{\prime }}_{IF-INS}(m)$	$\delta \Delta {\tilde{N}}_{1}^{'}(\mathrm{cycle})$	$\delta \Delta {\tilde{N}}_{2}^{'}(\mathrm{cycle})$
192200	1	0	0.860	0.483	1.00	0.00	0.862	0.485	1.00	0.01
192400	0	2	−1.723	−0.755	−0.01	1.99	−1.724	−0.756	−0.01	1.99
192600	2	1	0.877	0.600	2.02	1.00	0.862	0.585	1.94	0.94
192800	1	3	−1.698	−0.623	1.13	3.10	−1.724	−0.649	1.00	3.00
193000	3	3	−0.010# ^1^	0.311	2.95	2.96	0.000#	0.321	3.00	3.00
193200	2	4	−1.716	−0.539	1.99	3.98	−1.724	−0.547	1.95	3.94
193400	4	3	0.867	0.808	4.00	3.00	0.862	0.803	3.98	2.98
193600	4	5	−0.857	0.059#	4.06	5.06	−0.862	0.054	4.03	5.03
193800	5	3	1.729	1.292	5.00	2.99	1.724	1.287	4.97	2.97
194000	6	4	1.733	1.405	6.04	4.03	1.724	1.396	5.99	3.99
194200	7	0	6.034	3.393	7.02	0.02	6.034	3.393	7.01	0.02
194400	7	9	−1.731	−0.012#	6.97	8.98	−1.724	−0.005#	7.01	9.01
194600	8	7	0.873	1.240	8.02	7.00	0.862	1.229	7.96	6.96
194800	9	7	1.713	1.710	8.97	6.98	1.724	1.721	9.03	7.03

**^1^** # represents the failure detection of the cycle slip.

**Table 2:** Resolution of real cycle slips with signal interruption from 10 s to 60 s.

**PRN**	**Simulated CS**		**10 s Outage**				**20 s Outage**			
	**L1(cycle)**	**L2(cycle)**	$\delta \Delta {\tilde{N}}_{1}$	$\delta \Delta {\tilde{N}}_{2}$	$\delta \Delta {{\tilde{N}}^{\prime }}_{1}$	$\delta \Delta {{\tilde{N}}^{\prime }}_{2}$	$\delta \Delta {\tilde{N}}_{1}$	$\delta \Delta {\tilde{N}}_{2}$	$\delta \Delta {{\tilde{N}}^{\prime }}_{1}$	$\delta \Delta {{\tilde{N}}^{\prime }}_{2}$
06	1	0	1.13	0.08	0.90	–0.10	1.28* ^1^	0.19	0.90	–0.10
13	3	3	2.71*	2.77	3.01	3.01	2.56*	2.63*	2.88	2.88
16	5	4	5.20	4.15	4.97	3.98	5.48*	4.36*	4.92	3.92
19	7	9	6.86	8.88	6.92	8.92	6.69*	8.74*	6.90	8.89
23	42	9	41.65*	8.68*	41.72*	8.73*	41.50*	8.54*	41.66*	8.68*
27	60	77	60.04	77.00	59.90	76.89	60.12	77.06	59.87	76.86
**PRN**	**Simulated CS**		**30 s Outage**				**40 s Outage**			
	**L1(cycle)**	**L2(cycle)**	$\delta \Delta {\tilde{N}}_{1}$	$\delta \Delta {\tilde{N}}_{2}$	$\delta \Delta {{\tilde{N}}^{\prime }}_{1}$	$\delta \Delta {{\tilde{N}}^{\prime }}_{2}$	$\delta \Delta {\tilde{N}}_{1}$	$\delta \Delta {\tilde{N}}_{2}$	$\delta \Delta {{\tilde{N}}^{\prime }}_{1}$	$\delta \Delta {{\tilde{N}}^{\prime }}_{2}$
06	1	0	1.44*	0.31*	0.83	–0.17	1.68# ^2^	0.51#	0.91	–0.09
13	3	3	2.41#	2.51*	2.84	2.84	2.36#	2.48#	2.90	2.90
16	5	4	5.80#	4.59#	4.85	3.85	6.14#	4.85#	4.82	3.82
19	7	9	6.41#	8.49#	6.78	8.78	6.12#	8.27#	6.79	8.79
23	42	9	41.00#	8.15#	41.65*	8.66*	40.57#	7.80#	41.54*	8.55*
27	60	77	60.15	77.07	59.78	76.78	60.20	77.11	59.81	76.80
**PRN**	**Simulated CS**		**50 s Outage**				**60 s Outage**			
	**L1(cycle)**	**L2(cycle)**	$\delta \Delta {\tilde{N}}_{1}$	$\delta \Delta {\tilde{N}}_{2}$	$\delta \Delta {{\tilde{N}}^{\prime }}_{1}$	$\delta \Delta {{\tilde{N}}^{\prime }}_{2}$	$\delta \Delta {\tilde{N}}_{1}$	$\delta \Delta {\tilde{N}}_{2}$	$\delta \Delta {{\tilde{N}}^{\prime }}_{1}$	$\delta \Delta {{\tilde{N}}^{\prime }}_{2}$
06	1	0	1.87#	0.65#	0.90	–0.10	2.14#	0.85#	0.86	–0.14
13	3	3	2.44#	2.53*	2.85	2.85	2.43#	2.53*	2.88	2.88
16	5	4	6.58#	5.19#	4.84	3.84	7.04#	5.54#	4.78	3.78
19	7	9	5.75#	7.97#	6.76	8.76	5.28#	7.60#	6.74*	8.74*
23	42	9	40.21#	7.51#	41.51*	8.52*	39.66#	7.04#	41.33#	8.34#
27	60	77	60.25	77.13	59.76	76.76	60.30*	77.17	59.75	76.75

**^1^** * represents a resulting rounding error greater than ±0.25 cycles; ^2^ # represents an incorrect rounded cycle slip.
